# Editorial: Fibrotic Lung Disease—“Lumping” the Progressive Phenotype

**DOI:** 10.3389/fmed.2022.941008

**Published:** 2022-05-31

**Authors:** Tejaswini Kulkarni, Sydney B. Montesi, Bridget F. Collins

**Affiliations:** ^1^Division of Pulmonary, Allergy and Critical Care Medicine, The University of Alabama at Birmingham, Birmingham, AL, United States; ^2^Division of Pulmonary and Critical Care Medicine, Massachusetts General Hospital, Boston, MA, United States; ^3^Division of Pulmonary, Critical Care and Sleep Medicine, University of Washington Department of Medicine, Seattle, WA, United States

**Keywords:** progressive pulmonary fibrosis, interstitial lung diseases, idiopathic pulmonary fibrosis, antifibrotic therapy, immunosuppression

Interstitial lung diseases (ILD) comprise of a group of almost 200 entities characterized by heterogeneity in the extent of inflammation and/or fibrosis. Idiopathic pulmonary fibrosis (IPF), the prototypic fibrosing lung disease, is progressive and associated with significant mortality (1). A proportion of patients with non-IPF fibrosing ILD can present with progressive clinical behavior akin to IPF despite conventional therapies. While previously identified as progressive fibrosing ILD (PF-ILD), this cohort has been defined as progressive pulmonary fibrosis (PPF) per the recently published ATS/ERS/JRS/ALAT clinical practice guidelines (1). PPF is characterized by at least two of the three criteria: worsening of respiratory symptoms, physiological and/or radiological evidence of disease progression.

In this Research Topic, Case provides an overview of PPF highlighting specific ILDs that portend higher risk for disease progression. The most common ILDs with a higher proportion of PPF include idiopathic non-specific interstitial pneumonia, connective tissue disease associated ILDs, fibrotic hypersensitivity pneumonitis, unclassifiable ILD, fibrotic pneumoconiosis, and fibrotic sarcoidosis. Risk factors associated with developing PPF include male sex, older age, lower forced vital capacity and diffusion capacity for carbon monoxide at baseline and radiological or histological features of usual interstitial pneumonia (2–4).

There has been considerable progress in understanding the global impact associated of PPF. Cottin et al. conducted a structured literature review and describe the epidemiology as well as humanistic and economic burdens of PPF. The estimated prevalence of PPF ranges from 6.9 to 70.3/100,000 persons and the estimated incidence from 2.1 to 32.6/100,000 person-years globally from three reported studies (5–7). This wide range of estimates mainly results from variation in study design, geographical differences, and definitions of progression. Such variation may be mitigated in the future by the recently published PPF criteria (1). Along with reduced quality of life among patients with PPF, this review highlights the greater economic burden, with higher indirect costs including job losses, and higher healthcare utilization/related costs when compared with the overall non-IPF ILD population. This underscores the urgent need to conduct high-quality research for early identification and treatment of PPF.

As the concept of PPF evolves, there is increasing interest in biomarkers (medical signs that can be accurately and reproducibly measured to characterize a disease state or outcome) to aid in determining which patients with pulmonary fibrosis may have progressive disease and to guide diagnosis and treatment (8). In this Research Topic, Bowman et al. provide a review of biomarkers in PPF based on compartment: peripheral blood, airway, and pulmonary parenchyma. The authors highlight the need for future study, particularly of biomarkers that may distinguish inflammatory from fibrotic interstitial lung diseases. Some biomarkers such as elevated peripheral blood monocyte count (associated with increased mortality risk in IPF and other fibrotic lung diseases) are already widely available in clinical practice, but it is not yet known whether and how they should influence management (Bowman et al.).

High resolution computed tomography (HRCT) of the chest is a fundamental component of PPF diagnosis and evaluation. In this series, Qubo et al. review the role of HRCT in PFF diagnosis. While HRCT may aid in diagnosis and prognostication among patients with IPF and other fibrotic lung diseases, there is interobserver variability in assessment of features, pattern and interval change (9). Quantitative computed tomography (QCT) has been studied as a biomarker of fibrotic lung disease, although it is not yet clear how to best use QCT serially or at a single time point to predict disease course or response to therapy (9). Recently published guidelines on PPF note that additional validation and standardization of protocols are needed before QCT can be used widely (1).

There is growing recognition that despite differences in underlying ILD subtype, the clinical course of PPF is similar to IPF. This has resulted in the expansion of clinical trials of antifibrotics (pirfenidone and nintedanib) to include patients with PPF (10, 11), the FDA-approval of nintedanib for PF-ILD, and the inclusion of patients with PF-ILD alongside patients with IPF for evaluating new therapies (12). In this Research Topic, Copeland and Lancaster review key management aspects for patients with PFF ranging from pharmacologic therapies, such as use of nintedanib, to treatment of common co-morbidities, such as pulmonary hypertension or gastroesophageal reflux disease, to the importance of non-pharmacologic therapies such as pulmonary rehabilitation. The authors highlight the importance of early referral to lung transplantation for appropriate patients and the role of palliative care for management of symptom burden. It should be noted that outside of data from the INBUILD and RELIEF trials (10, 11) most evidence related to management considerations for patients with PPF is based on data obtained in an IPF population.

While considerable advances have been made in our approach to treating PPF, there remain important unanswered questions particularly surrounding therapeutic management. First, patients with PPF are often treated with immunosuppression depending on the underlying ILD subtype. How effective immunosuppression is in patients once a progressive non-IPF fibrotic phenotype has become evident remains to be determined. Based on current treatment guidelines, initiation of antifibrotic therapy, specifically nintedanib, is recommended when conventional treatment for PPF has failed (1). Whether patients with PPF would benefit from a more “upfront” approach to initiation of antifibrotic therapy has not been studied. Additionally, the “lumping” of the progressive phenotype may fail to capture underlying disease heterogeneity by subtype that could influence response to treatment. As study and validation of biomarkers evolves, precision medicine will likely play a role not only in diagnosis but also in treatment of PPF. This is the basis for the ongoing PRECISIONS IPF study (NCT 04300920), enrolling IPF patients who screen positive for a particular polymorphism in the TOLLIP gene to determine treatment effect of N-acetylcysteine. Such an approach will likely be essential in treatment of PPF, particularly as effects of medications such as immunomodulators may vary based on biomarkers such as leukocyte telomere length and across subtypes of disease (13). This series highlights progress made in understanding PPF that paves the way for further investigations to identify best management practices for these patients.

## Author Contributions

TK, SM, and BC wrote sections of the manuscript. All authors contributed to manuscript revision, read, and approved the submitted version.

## Conflict of Interest

TK has received speaker fees from Boehringer Ingelheim Pharmaceuticals, Inc. and consulting fees from Puretech, Inc. and United Therapeutics Inc., outside the submitted work. SM was funded by NIH/NHLBI (K23HL15033) and reports research funding from Pliant Therapeutics, Merck, United Therapeutics, and Boehringer Ingelheim, consulting fees from DevPro Biopharma, Gilead Sciences, and Roche, and royalties from Wolters Kluwer, outside the submitted work. BC has received consulting fees from Boehringer Ingelheim Pharmaceuticals, Inc, outside the submitted work.

## Publisher's Note

All claims expressed in this article are solely those of the authors and do not necessarily represent those of their affiliated organizations, or those of the publisher, the editors and the reviewers. Any product that may be evaluated in this article, or claim that may be made by its manufacturer, is not guaranteed or endorsed by the publisher.
